# Gelatin-chondroitin-6-sulfate-hyaluronic acid scaffold seeded with vascular endothelial growth factor 165 modified hair follicle stem cells as a three-dimensional skin substitute

**DOI:** 10.1186/scrt508

**Published:** 2014-10-20

**Authors:** Renfu Quan, Xuan Zheng, Shichao Xu, Liang Zhang, Disheng Yang

**Affiliations:** Research Institute of Orthopedics, Xiaoshan Traditional Chinese Medical Hospital, 156 Yucai Road, Zhengv Jiang Province, 311200 China; Research Institute of Orthopedics, Zhejiang Chinese Medical University, Binwen Road, Hangzhou, Zhejiang Province China; Research Institute of Orthopedics, The Second Affiliated Hospital, Medical College of Zhejiang University, Jiefang Road, Hangzhou, Zhejiang Province China

## Abstract

**Introduction:**

In the field of skin tissue engineering, gelatin-chondroitin-6-sulfate-hyaluronic acid (Gel-C6S-HA) stents are a suitable bio skin substitute. The purpose was to investigate the effect of genetically-modified hair follicle stem cells (HFSCs), combined with Gel-C6S-HA scaffolds, on the vascularization of tissue-engineered skin.

**Methods:**

Three-dimensional (3D) Gel-C6S-HA scaffolds were prepared by freeze-drying. Vascular endothelial growth factor (VEGF) 165 gene-modified rat HFSCs (rHFSCs) were inoculated into the scaffolds and cultured for 7 days. Two bilateral full-thickness skin defects were created on the back of 18 Sprague–Dawley rats. Rats were randomly divided into four groups: Group A, HFSCs transduced with VEGF165 seeded onto Gel-C6S-HA scaffolds; Group B, HFSCs transduced with empty vector seeded onto Gel-C6S-HA scaffolds; Group C, Gel-C6S-HA scaffold only; Group D, Vaseline gauze dressing. These compositions were implanted onto the defects and harvested at 7, 14 and 21 days. Wound healing was assessed and compared among groups according to hematoxylin-eosin staining, CD31 expression, alpha smooth muscle actin (α-SMA) and major histocompatibility complex class I (MHC-I) immunohistochemistry, and microvessel density (MVD) count, to evaluate the new blood vessels.

**Results:**

SEM revealed the Gel-C6S-HA scaffold was spongy and 3D, with an average pore diameter of 133.23 ± 43.36 μm. Cells seeded on scaffolds showed good adherent growth after 7 days culture. No significant difference in rHFSC morphology, adherence and proliferative capacity was found before and after transfection *(P* >0.05). After 14 and 21 days, the highest rate of wound healing was observed in Group A (*P* <0.05). Histological and immunological examination showed that after 21 days, MVD also reached a maximum in Group A (*P* <0.05). Therefore, the number of new blood vessels formed within the skin substitutes was greatest in Group A, followed by Group B. In Group C, only trace amounts of mature subcutaneous blood vessels were observed, and few subcutaneous tissue cells migrated into the scaffolds.

**Conclusions:**

Tissue-engineered skin constructs, using 3D Gel-C6S-HA scaffolds seeded with VEGF165-modified rHFSCs, resulted in promotion of angiogenesis during wound healing and facilitation of vascularization in skin substitutes. This may be a novel approach for tissue-engineered skin substitutes.

## Introduction

Large skin defects caused by trauma often result in severe physical disability and even death. Current treatment methods include wound dressings, autologous skin grafts, allogeneic skin grafts and tissue-engineered skin repair, to name a few. However, limitations exist for these approaches. For example, wound dressings have no physiological function, autologous skin grafts have limited area coverage and allogeneic skin grafts often lead to an immunological rejection response, probably skin shedding and necrosis, which could lead to secondary damage in the patient and increased morbidity. Application of tissue-engineered skin could potentially resolve many of these limitations. Current tissue-engineered skin repair approaches are often complicated by wound infection, nonunion and other complications, and treatment efficacy is unsatisfactory. This is closely related to the extent of vascularization of the repaired wound [1–3]. Poor angiogenesis capability can lead to a limited vascular system and insufficient supply of nutrients to the early grafted skin, which in turn can lead to necrosis of the skin substitute and graft failure. Facilitation of the process of vascularization is thus an unmet clinical need in the field of skin tissue engineering [4–6]. Composite delivery system construction [7, 8], screening of cell-seed types [9–12], incorporation of effective active factors [13, 14] and applying genetically-modified cells [10–12] can promote early vascularization of tissue-engineered skin. Gelatin–chondroitin-6-sulfate–hyaluronic acid (Gel-C6S-HA) scaffolds are known to be hydrophilic with good tissue compatibility and biodegradability [15, 16]. Gelatin, a denatured collagen, is nontoxic, is biocompatible and can provide a microenvironment for adherence, growth, proliferation and differentiation of cells. Incorporation of chondroitin-6-sulfate into gelatin scaffolds resulted in a scaffold with higher resistance to collagenase degradation, higher elastic modulus and a more porous structure than gelatin scaffolds [17, 18]. It can significantly enhance the flexibility and porous structure of the scaffold. As the strongest natural moisturizing factor and when used at a certain concentration, hyaluronic acid can effectively improve scaffold strength and the *in vivo* degradation rate, prevent drying of the scaffold and provide nutrients to the cells within the scaffold [18, 19]. In the tissues of the skin, this characteristic is of fundamental importance for water retention. Hyaluronic acid can be further modified by hydroxyl and carboxyl functional groups with specific cell or extracellular matrix components, to enhance its biological function [20].

Hair follicle stem cells (HFSCs) are undifferentiated cells with fast self-renewing potential and rapid *in vitro* proliferative capacity, localized mainly in the bulge of the hair follicle outer root sheath [21, 22]. Studies have shown that cultured HFSCs have high colony-forming ability and very high regenerative potential [23]. HFSCs not only can differentiate into hair follicle cells, but also into nerve cells, melanoma cells, smooth muscle cells and epithelial cells, to name a few. HFSCs can be harvested from follicle skin and hair, and their numbers are extremely impressive. HFSC harvesting poses no serious complications and provides the most readily available source of stem cells [24–27].

As a specific vascular endothelial cell mitogen, vascular endothelial growth factor (VEGF) plays an important role in angiogenesis and the repair process after tissue ischemia. There are five subtypes of VEGF, with VEGF165 being the most active, widely distributed and main active form in the body [28, 29]. However, VEGF165 has a very short half-life and can be easily diluted after injection into the body. Shima and colleagues reported that the biological half-life of VEGF165 is 30 to 45 minutes under normal oxygen partial pressure, and 6 to 8 hours under hypoxia [30]. Adding exogenous VEGF165 into tissue-engineered skin therefore has limited therapeutic efficacy. To overcome the limitations of pure protein treatment, application of gene therapy should be considered.

The purpose of this study was to analyze the effects of genetically-modified HFSCs combined with Gel-C6S-HA scaffolds on the vascularization of skin substitutes. First, HFSCs with high proliferative capacity were obtained using double digestion with dispase and type IV collagenase. Cells were then screened by microisolation and differential adherence of type IV collagen. HFSCs were then genetically modified using lentivirus-mediated VEGF165, after which sustained and stable expression of VEGF165 at high abundance was examined. Rat hair follicle stem cells (rHFSCs) were then inoculated into Gel-C6S-HA scaffolds and cultured for 7 days. rHFSC morphology, adherence and proliferation were observed. Finally, the constructed tissue-engineered skin was grafted onto the back of rats with full-thickness skin defects to evaluate angiogenesis potential, wound healing and immunogenicity of the composite scaffolds at different time points.

## Methods

### Preparation of Gel-C6S-HA scaffolds

Gelatin (5% (w/v); Sigma, San Jose, CA, USA) was dissolved in 10 ml distilled water and slowly added to chondroitin-6-sulfate (0.05% (w/v); Sigma) and hyaluronic acid (0.1% (w/v); Sigma) to form a suspension solution. The solution was stirred for 60 minutes at room temperature with a magnetic stirrer, and then cross-linker solution (0.5% (w/v) 1-ethyl-3-(3-dimethylaminopropyl) carbodiimide and 0.25% (w/v) *N*-hydroxysuccinimide; Sigma) was added dropwise to the suspension solution and stirred for 15 minutes. The mixture was injected into wells of a cell culture plate (2 cm diameter, 1.8 cm height; Corning-costar, NY, USA). Plates were agitated horizontally to enable even cell distribution. Constructs were then frozen at -80°C for 2 hours and lyophilized with a freeze dryer (CHRIST, Vaihingen, Germany) for 48 hours. Gel-C6S-HA porous sponge-like scaffolds, with a thickness of 2 mm, were obtained. Scaffolds were soaked in 75% (v/v) ethanol followed by phosphate-buffered saline (PBS), each for 48 hours, and were dried with sterile gauze for further use. The macroscopic appearance of the Gel-C6S-HA scaffold was photographed using a digital camera (Sony, Tokyo, Japan).

### Isolation and culture of rat HFSCs

Sprague–Dawley rats were provided by the Experimental Animal Center of Zhejiang Chinese Medical University (batch number: SCXK (Zhejiang) 2013‒0023). The experimental protocol was approved by the Experimental Animal Ethics Committee of Zhejiang Chinese Medical University, and animal disposal was in line with animal ethics requirements. Two Sprague–Dawley rats (1 week old) were euthanized by cervical dislocation. The skin near the beards was cut with ophthalmic scissors, rinsed (3×) in PBS and digested with a mixture of 1% (w/v) dispase (Gibco, GrandIsland, NY, USA) and 1% (w/v) type IV collagenase (Gibco) at 37°C for 90 minutes, prior to further PBS rinsing (3×). Hair follicles were isolated from the connective tissue sheath using stereomicroscopy and a needle. The two ends were cut and the bulge was inoculated in a plastic dish pre-coated with Matrix gel (Gibco), prior to adding 1 ml medium. The medium components comprised: 86.896% (v/v) Dulbecco’s modified Eagle’s medium/F12 medium (Gibco), 10% (v/v) Knockout™ Serum Replacement (Gibco), 1% (v/v) penicillin–streptomycin mixture (Solarbio, Beijing, China), 1% (v/v) l-glutamine (Gibco), 1% (v/v) nonessential amino acids (Gibco), 0.002% (v/v) epidermal growth factor (Becton, Dickinson and Company, Franklin Lakes, NJ, USA), 0.001% (v/v) basic fibroblast growth factor (Becton, Dickinson and Company), 0.1% (v/v) hydroxyl ethanol (Gibco) and 0.001% (v/v) hydrocortisone (Sangon Biotech, Shanghai, China). HFSCs were cultured at 37°C, 5% (v/v) carbon dioxide for 2 days. Medium (5 ml) was added following tissue adherence and changed every 3 days thereafter. Cell migration and growth conditions were then observed.

The Petri dish was coated with type IV collagen (Sigma, AL, St. Louis, MO, USA) and left for 1 hour at room temperature. The primary cells were digested with TrypLE™ Express trypsin substitute enzyme (Gibco) and inoculated in culture dishes. Nonadherent cells and medium were aspirated after 20 minutes and adherent cells were further cultured in the medium. Passage (P) 2 generation cells were further purified as well as the primary rHFSCs.

### Characterization of relevant genes by quantitative polymerase chain reaction

Quantitative polymerase chain reaction (PCR) was carried out on purified rHFSCs (P3) as follows. Six target genes (cytokeratin (CK) 10, CK15, CD34, CK19, integrin β1, integrin α6) and one internal reference gene (beta-actin (ACTB)) were amplified in a reaction tube. Primers were designed using Premier 5.0 software (Primer, Toronto, Canada) and the primer information is presented in Table 1. The PCR reaction included 10 μl of 2× SYBR Green Mix, 1 μl Primer Mix, 1 μl template and 8 μl ultrapure water. The reaction mixture was dispensed in a PCR eight-tube and mixed well. Quantitative PCR was carried out using a fluorescent quantitative PCR instrument (Bio-Rad, Hercules, CA, USA) with the SYBR Green method. The relative expression of mRNA for each gene was measured using the ∆Ct method:

**Table 1:** **Primer sequences used for reverse transcription–polymerase chain reaction gene expression analysis**

Gene	5′ to 3′	Primers	Production size (base pairs)
CK10	Forward	TTGGAAACCTGCAAATAACCC	175
	Reverse	ATCATAGACGAAAGGACTCTACCC	
CK15	Forward	AAAACCGTCGGGATGTAGAGG	94
	Reverse	TTGCTGGTCTGGATCATTTCTGT	
CK19	Forward	CCAAGTTTGAGACAGAACAGGC	156
	Reverse	CGTGGTTCTTCTTCAGGTAGGC	
CD34	Forward	CCTGCCGTCTGTCAATGTTTC	146
	Reverse	GCACTCCTCGGATTCCTGAAC	
Integrin β1	Forward	ATCATGCAGGTTGCAGTTTG	72
	Reverse	CGTGGAAAACACCAGCAGT	
Integrin α6	Forward	CGTGGTTCTTCTTCAGGTAGGC	188
	Reverse	CACATCTATGGACGCCCTCAC	
ACTB	Forward	GCTATGTTGCCCTAGACTTCGA	173
	Reverse	GATGCCACAGGATTCCATACC	
VEGF165	Forward	CACCCACCCACATACATACA	169
	Reverse	CTCCCAACTCAAGTCCACA	
β-actin	Forward	GTCCCTCACCCTCCCAAAAG	20
	Reverse	GCTGCCTCAACACCTCAACCC	21

### Characterization of cells using immunofluorescence staining

rHFSCs (P3) were inoculated on a slide and cultured for 2 days. Cells were rinsed with PBS–Tween, fixed with 4% (w/v) paraformaldehyde (Kelong Chemical Reagent Company, Chengdu, China) and blocked with 5% (w/v) bovine serum albumin at room temperature. Integrin β1 (1:100; Abcam, Cambridge, UK), integrin α6 (1:50; Abcam) and CK15 (1:100; Abcam) antibodies were then added separately. PBS–Tween was added instead of primary antibody in the control group. After incubation at room temperature and washing with PBS–Tween, fluorescein-labeled secondary antibody was added (1:100; Jackson, San Francisco, CA, USA) and incubated in the dark for 30 minutes. 4′,6-Diamidino-2-phenylindole (1:2,000 dilution; Roche, La Roche, Switzerland) was then added and incubated for 5 minutes for nuclear staining. Cells were then air dried in the dark, mounted with Mounting Solution and observed using fluorescence microscopy (Olympus, Tokyo, Japan).

### Cell proliferation measurement

rHFSCs (P3, P5, P7 and P9) with good growth status were inoculated in a dish at a concentration of 1 × 10^5^ cells/well. Cells were counted at days 1, 2, 3, 4, 5, 6 and 7 with a hemocytometer. The growth curve was plotted from an average of six replicates.

### Modification of rHFSCs with lentiviral gene

#### Package of lentivirus

A calcium phosphate transfection kit (Biowit Technologies, Shengzhen, China) was used to package the lentivirus. Cells (293 T) were inoculated 24 hours in advance and grown to 50 to 70% confluence. Before transfection, medium was replaced with high-glucose Dulbecco’s modified Eagle’s medium (Gibco) +10% (v/v) fetal bovine serum (Gibco) without antibiotics. The target plasmid (pLV-VEGF165–internal ribosome entry site–enhanced green fluorescent protein) and packaging plasmids (vesicularstomatitisvirusG (VSVG), Respiratory Syncytial Virus - Respiratory Entericorphan Virus (RSV-REV) and Rev response element (RRE)) were added to Hank’s balanced salt solution (Gibco) at a ratio of 2:1:1:1 (w/w). Components were mixed well and supplemented with double-distilled water. The mixture was referred to as Solution A. After addition of CaCl_2_, the solution was left to sit at room temperature for 20 minutes, prior to adding dropwise to a cell culture dish. After 10 to 12 hours, medium comprising high-glucose Dulbecco’s modified Eagle’s medium +10% (v/v) fetal bovine serum +1% (v/v) penicillin–streptomycin was added. After 48 hours, while strong green fluorescence was expressed, the supernatant was collected and stored at -80°C for further use. Packaging of pLV–internal ribosome entry site–enhanced green fluorescent protein plasmid was carried out using identical steps.

### Transfection of rHFSCs with lentivirus

rHFSCs were cultured with 50 μl virus in an incubator at 37°C with 5% (v/v) carbon dioxide for 30 minutes. Additional medium was then added and the cells were further cultured. Expression of green fluorescence was observed using a fluorescence microscope (Olympus) after 72 hours. Fields of view (12 × 200) were randomly observed to calculate the transfection efficiency:


The average was calculated from three replicates. Cells were observed again at day 14.

### Reverse transcription-polymerase chain reaction

Total RNA was extracted using Trizol RNA extraction kit (Kang Century, Shanghai, China), according to the manufacturer’s instructions. cDNA was obtained by adding reverse transcriptase according to the manufacturer’s instructions. Primers were designed using Premier5.0 software, and the primer information is presented in Table 1. The PCR reaction mixture included 1 μl template, 2 μl of 10 × PCR buffer, 1 U Taq DNA polymerase, 0.5 μl each 5′ and 3′ primer, and the volume was brought up to 20 μl with ultrapure water. The product was run on a 2% (w/v) agarose gel for 30 minutes. Imaging was obtained and developed using an imaging system (Bio-Rad, San Francisco, CA, USA).

### Western blot

Cells were rinsed with cold PBS (3×) and proteins were extracted on ice. Proteins were run on a sodium dodecyl sulfate polyacrylamide electrophoresis gel and transferred to a polyvinylidene fluoride membrane. The membrane was soaked with 5% (w/v) skimmed milk for 1 hour, and then blocked in blocking solution overnight at room temperature. After rinsing the membrane in PBS (3×), VEGF165 antibody (1:2,000; R&D Systems, Minneapolis, MN, USA) was added and incubated for 1 hour. After rinsing with Tris-buffered saline–Tween (3×), goat anti-rabbit secondary antibody (1:1,000; Jackson, West Grove, PA, USA) was added and incubated at room temperature for 1 hour. After washing the membrane with Tris-buffered saline–Tween (3×), electrochemiluminescence (ECL) reagent was added, color was developed using an odyssey machine (LI-COR, Lincoln, NE, USA) and images were obtained.

### Cell morphology, adherence and proliferation on scaffolds

rHFSCs were digested into single-cell suspensions prior to inoculation in Gel-C6S-HA scaffolds (Groups A, B and C) at a density of 5 × 10^6^/cm^2^ and were gas–liquid incubated at 37°C with 5% (v/v) carbon dioxide. After 1 and 7 days, cell-seeded scaffolds were fixed in 2.5% (v/v) glutaraldehyde solution overnight, prior to fixing with 1% (v/v) osmium tetroxide for 1 hour. After a PBS rinse (3×), constructs were dehydrated with gradient acetone 50 to 100% (v/v). Following critical point drying (Leica, Osaka, Japan), samples were sputter-coated with gold and observed using scanning electron microscopy (Hitachi, Tokyo, Japan) at a voltage of 15 kV.

Proliferation was measured using the CCK-8 Kit (Qcbio Science & Technologies, Shanghai, China). rHFSCs were inoculated on the three-dimensional scaffolds. At 1, 3, 5 and 7 days, 10% (v/v) CCK-8 was added to the medium and incubated for 4 hours. Optical density at 450 nm was measured using a microplate reader (BioTek, Winooski, VT, USA).

### Grafting of composite scaffolds

Rats were injected intraperitoneally with 1% (w/v) sodium pentobarbital (40 mg/kg), preoperatively, with fixed limbs. Rat backs were disinfected with povidone–iodine and the operative areas were treated for hair removal. Skin was incised along marked lines, deep to the subcutaneous superficial fascia layer, and the full-thickness skin was removed. Four 1.2 cm × 1.2 cm wounds were opened 1 cm from the dorsal midline, two on each side, spaced by 1 cm. The rats were randomly divided into three batches and four groups: Group A (experimental group), HFSCs/Gel-C6S-HA scaffold transfected with VEGF165; Group B, HFSCs/Gel-C6S-HA scaffold transfected with empty vector; Group C, Gel-C6S-HA scaffold; and Group D, Vaseline gauze (Zhengde Surgical Dressing Company, Shaoxing, China). Materials for all four groups were grafted into the wounds, which were then interrupted sutured with 5–0 silk thread at the wound edge, covered with sterile dressing, and fixed by strapping. To prevent the rat biting the wound area, a resilient protection coat was designed and applied.

For postoperative treatment, each rat was housed in a separate cage. The outer layer of the surgical dressings was soaked and replaced immediately with sterile gauze. Sodium penicillin (1,000,000 U/kg; North China Pharmaceutical Company, Shanghai, China) was injected daily.

### Observation of postoperative wound

At 7, 14 and 21 days after grafting, wounds were photographed using a digital camera (Sony, Tokyo, Japan) and the rate of wound healing was calculated using Image-Pro Plus 6.0 image analysis software (Media Cybernetics, Rockville, MD, USA):


When the majority of the graft was absorbed and tightly combined with the surrounding wound, this was considered as healed.

### Tissue sections

Fresh samples were rinsed with saline, fixed with 4% (w/v) paraformaldehyde solution, embedded in paraffin and sliced (6 μm). Slices were dehydrated with gradient ethanol, stained with hematoxylin and eosin, treated with xylene and mounted with neutral balsam. Each inverted slice was observed using phase contrast microscopy (Olympus).

### Immunological examination

Fixation, embedment and slicing were carried out as described above. After dewaxing, hot antigen retrieval was performed in 0.01 M citrate buffer solution for 15 minutes. Samples were blocked with 8% (w/v) bovine serum albumin, then incubated with anti-CD31 (1:100; Abcam) and anti-alpha smooth muscle actin (anti-α-SMA, 1:150; Abcam) antibodies overnight at 4°C, separately. After washing with PBS (3×), horseradish-peroxidase-labeled secondary antibody (1:200; Jackson) was added and incubated at room temperature for 2 hours followed by PBS washing (3×). 3,3′-Diaminobenzidine solution was then added to each sample and incubated for 20 minutes to develop the color. After mounting, inverted slices were observed using phase contrast microscopy.

### Microvessel density measurement

Immunohistochemical staining for CD31 was carried out on Groups A to C postoperatively at 7, 14 and 21 days. Brown dots present in images of endothelial cells indicated positive staining. Six random, unrepeated fields were selected for observation (×400). The number of newly grown microvessels (brown staining) was calculated using Image-Pro Plus 6.0, according to the conversion of each vision field area of 0.1885 mm^2^ being equal to 1 mm^2^. An average of six replicates was recorded as the microvessel density (MVD).

### Immunogenicity examination

Fixation, embedment, slicing, dewaxing and antigen retrieval methods were carried out as described above. Briefly, samples were blocked with 8% (w/v) bovine serum albumin overnight at 4°C. Histocompatibility antibody major histocompatibility complex class I (MHC-I, 1:20; Abcam) was added and incubated at 4°C overnight. After washing with PBS (3×), fluorescein-labeled secondary antibody (1:50; Jackson) was added and incubated at room temperature for 1 hour. After washing with PBS (3×), 4′,6-diamidino-2-phenylindole was added for nuclei staining. Samples were observed using fluorescence microscopy (Olympus) after mounting.

### Statistical analysis

Differences between groups were analyzed using the SPSS version 18.0 least significant difference test (SPSS Inc. Chicago, IL, USA). *P* <0.05 indicates a statistically significant difference.

## Results

### Morphology of three-dimensional Gel-C6S-HA scaffolds

**Figure 1:**
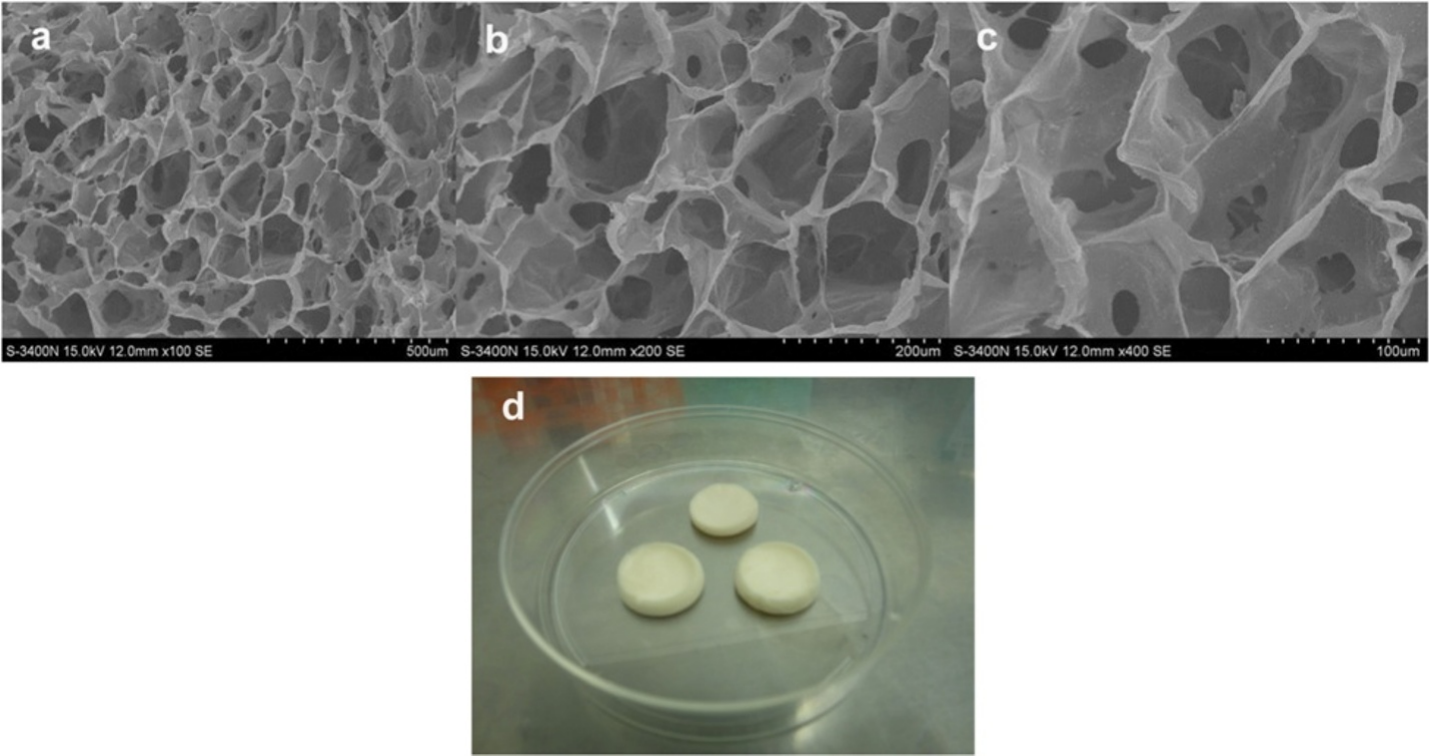
**Observation of Gel-C6S-HA scaffold characteristics. (a)**, **(b)** The scaffold formed a three-dimensional sponge-like structure, with connective transport holes between pores, observed using scanning electron microscopy. Pores were circular or polygonal in microstructure. **(c)** Pore sizes were not uniform, with an average pore diameter of 133.23 ± 43.36 μm. **(d)** Macroscopic appearance of the gelatin–chondroitin-6-sulfate–hyaluronic acid (Gel-C6S-HA) scaffold.

Gel-C6S-HA scaffolds made from freeze-drying were observed using scanning electron microscopy (Figure 1). By applying different magnifications, scaffolds were observed to form a spongy three-dimensional structure with transport holes between pores, which were circular or polygonal (Figure 1a,b). Using Image-Pro Plus 6.0 software, the average pore diameter was calculated to be 133.23 ± 43.36 μm, with varying sizes (Figure 1c). The macroscopic appearance of the Gel-C6S-HA scaffold is showed in Figure 1d.

### Primary culture of rat HFSCs and their biological characteristics

**Figure 2:**
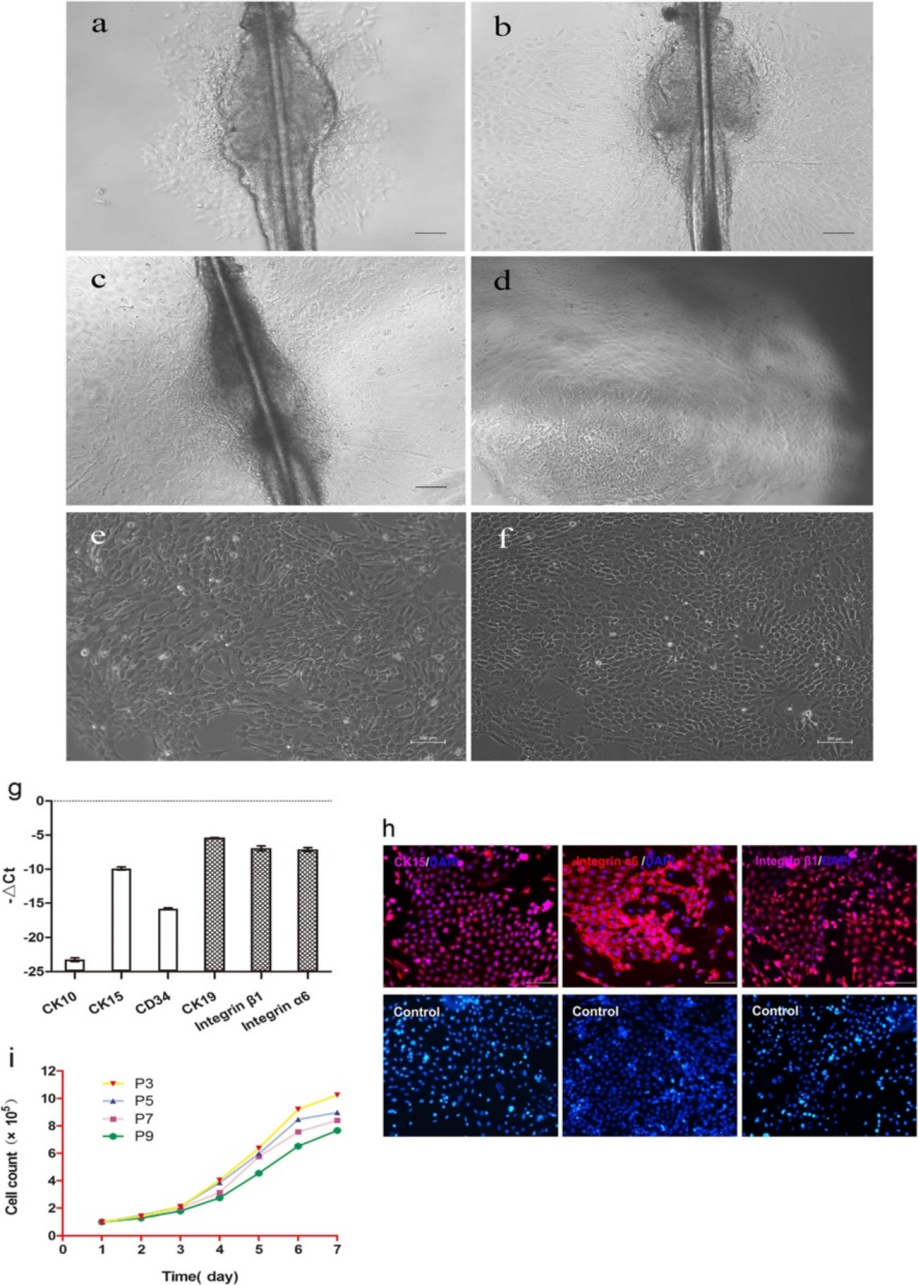
**Isolation and biological characteristics of rat hair follicle stem cells.** Primary cell culture on **(a)** day 3, **(b)** day 7 and **(c)**, **(d)** day 14. Rat hair follicle stem cells (HFSCs; P2) **(e)** before and **(f)** after purification. **(g)** Quantitative polymerase chain reaction results of six correlated genes in rat HFSCs; ACTB was used as the reference gene. **(h)** Immunofluorescence staining for expression of cytokeratin (CK) 15, integrin α6 and integrin β1. **(i)** Growth curves of different generations of HFSCs. Scale bars: 100 μm **(a to c, e, f, h)**; 250 μm **(d)**. Ct, cycle threshold; P, passage.

By 3 days, a small number of cells had migrated from the periphery of the follicle bulge (Figure 2a). By 7 days, the number of cells had gradually increased (Figure 2b). On 14 days, excess tissue was removed and adherent cells were observed to be tightly packed on the vessel wall, typical of epithelial cells (Figure 2c,d). After type IV collagen sorting of adherent cells (twice), cells showed typical cobblestone-like and nest-like morphology, with a clear three-dimensional appearance and high refractive index. Furthermore, cells aggregated and formed colonies, had a central cytoplasm and had round nuclei, the latter of which were large and prominent (Figure 2e,f).HFSCs (P3) were detected for expression of CK10, CK15, CK19, CD34, integrin α6 and integrin β1 genes. Expression levels of CK19, integrin α6 and integrin β1 genes were high, with moderate CK15 expression and low expression for CD34 and CK10 (Figure 2g). Immunological staining identified positive expression for CK15 (red florescence in cytoplasm), integrin α6 and integrin β1 in HFSCs. Conversely, expression of these markers was negative in the control group, which was counterstained with 4′,6-diamidino-2-phenylindole (Figure 2h).Cell growth curves were used to determine the proliferative capacity of the HFSCs (Figure 2i). At P3, P5, P7 and P9, HFSCs were in the interphase period within 2 days after inoculation and grew slowly. By 3 days, stem cell clones formed. From 5 to 6 days, cells were in the logarithmic growth phase with relatively rapid cell proliferation, after which the cell growth rate began to slow down, entering into a plateau period. From the seventh generation (P7 cells), cell proliferation gradually decreased.

### Genetic modification of rat HFSCs by VEGF165

**Figure 3:**
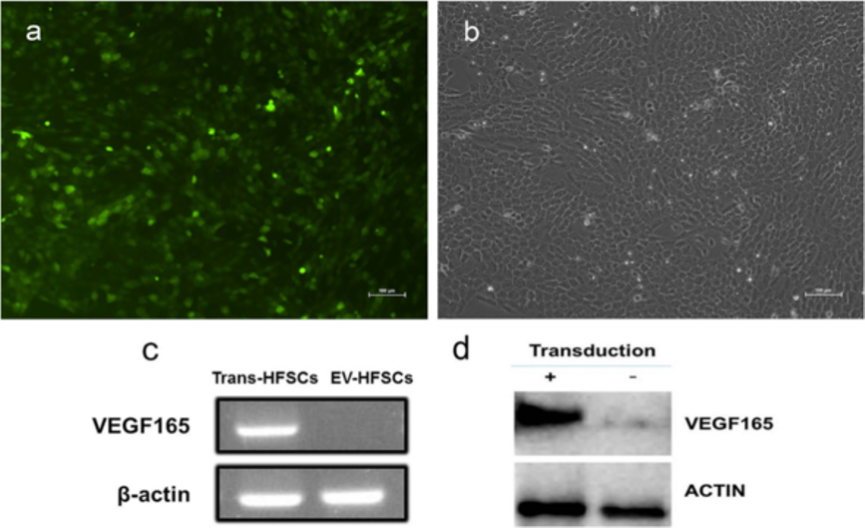
**VEGF165 gene-modified rat hair follicle stem cells. (a)** Fluorescein isothiocyanate image using fluorescence microscopy. Green staining indicates that the target plasmid pLV–VEGF165–IRES–EGFP was successfully transfected into the hair follicle stem cells (HFSCs). **(b)** Phase contrast image. **(c)** Reverse transcription-polymerase chain reaction for VEGF165 expression after transfection. **(d)** Western blot for expression of VEGF165 protein. Scale bars: 100 μm **(a, b)**. EGFP, enhanced green fluorescent protein; IRES, internal ribosome entry site; VEGF, vascular endothelial growth factor.

Strong green fluorescence was visible 72 hours after transfection with lentivirus (Figure 3a,b). The transfection efficiency was 71.52 ± 1.83%. Cells were continuously observed for 14 days, and the lentiviral transfection efficiency was steady at 85.76 ± 1.91%.Reverse transcription-PCR (Figure 3c) showed the VEGF165 mRNA expression level was significantly higher in the VEGF165 transfection group compared with the empty vector transfection group. Western blot results (Figure 3d) showed that VEGF165 protein was highly expressed in the VEGF165 transfection group, but not in the empty vector transfection group.

### Rat HFSC morphology, adherence and proliferation ability on scaffolds

**Figure 4:**
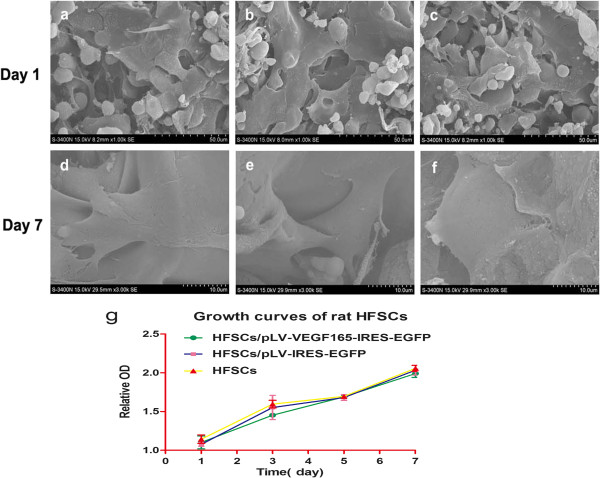
**Hair follicle stem cell morphology, adherence and proliferation on scaffolds. (a)**, **(b)**, **(c)** After 1 day of culture, no significant difference was observed between all groups with respect to cell morphology, with few adherent cells, the majority being spherical. **(d)**, **(e)**, **(f)** After 7 days of culture, all cells were firmly adhered to the scaffold and were growing three-dimensionally along the scaffold. **(g)** After transfection of VEGF165, hair follicle stem cells (HFSCs) adhered efficiently to the scaffold wall. There was no significant difference in proliferative capacity with the control group. **(a)**, **(d)** HFSCs after VEGF165 transfection (Group A). **(b)**, **(e)** Control group with empty support (Group B). **(c)**, **(f)** Control group (Group C). HFSCs/pLV-VEGF165-IRES-EGFP, HFSCs transduced with VEGF165 seeded on Gel-C6S-HA scaffolds; HFSCs/pLV-IRES-EGFP, HFSCs transduced with empty vector seeded on Gel-C6S-HA scaffolds: HFSCs, HFSCs seeded on Gel-C6S-HA scaffolds. EGFP, enhanced green fluorescent protein; Gel-C6S-HA, gelatin–chondroitin-6-sulfate–hyaluronic acid; IRES, internal ribosome entry site; OD, optical density; VEGF, vascular endothelial growth factor.

By 1 day, few adherent cells were observed in Groups A to C and the cells were spherical (Figure 4a,b,c). By 7 days, scanning electron microscopy was used to observe cell spreading and firm adherence to the scaffold walls (Figure 4d,e,f). CCK-8 showed there was no significant difference in the proliferative capacity of cells within the three groups (Figure 4g; *P* >0.05), indicating the scaffold had very good biocompatibility and was nontoxic. Taken together, gene-modified rHFSCs could adhere and grow well in the wall of the scaffolds.

### Transplantation of cell-seeded scaffolds

**Figure 5:**
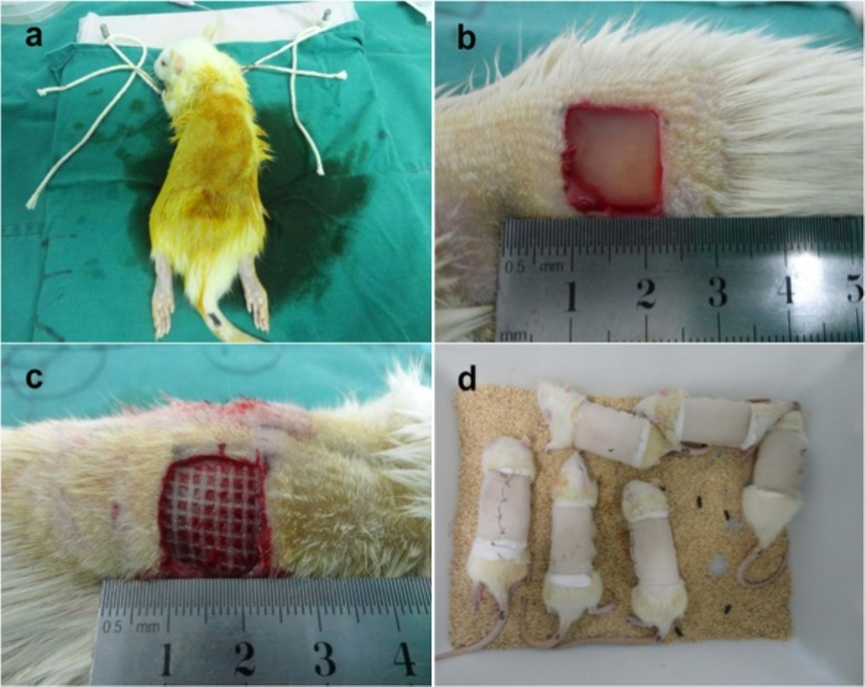
**Transplantation of the cell-seeded scaffold. (a)** After anesthesia with 1% (w/v) sodium pentobarbital, disinfection was carried out with iodine and hair removal treatment was performed in the surgical area. **(b)**, **(c)** The skin subcutaneous superficial fascia was incised and the skin substitute transplanted. **(d)** The specially designed flexible protective coat was used to prevent damage to the affected area from rat bites, preventing contamination and destruction.

After anesthesia, hair removal was carried out in the surgery zone on the back of the mice, as shown in Figure 5a. The four skin substitute groups were transplanted (Figure 5b,c) after sufficient hemostasis. By applying the designed elastic coat (Figure 5d), the skin was effectively protected from rat biting to reduce infection and destruction.

### Observation of postoperative skin wounds

**Figure 6:**
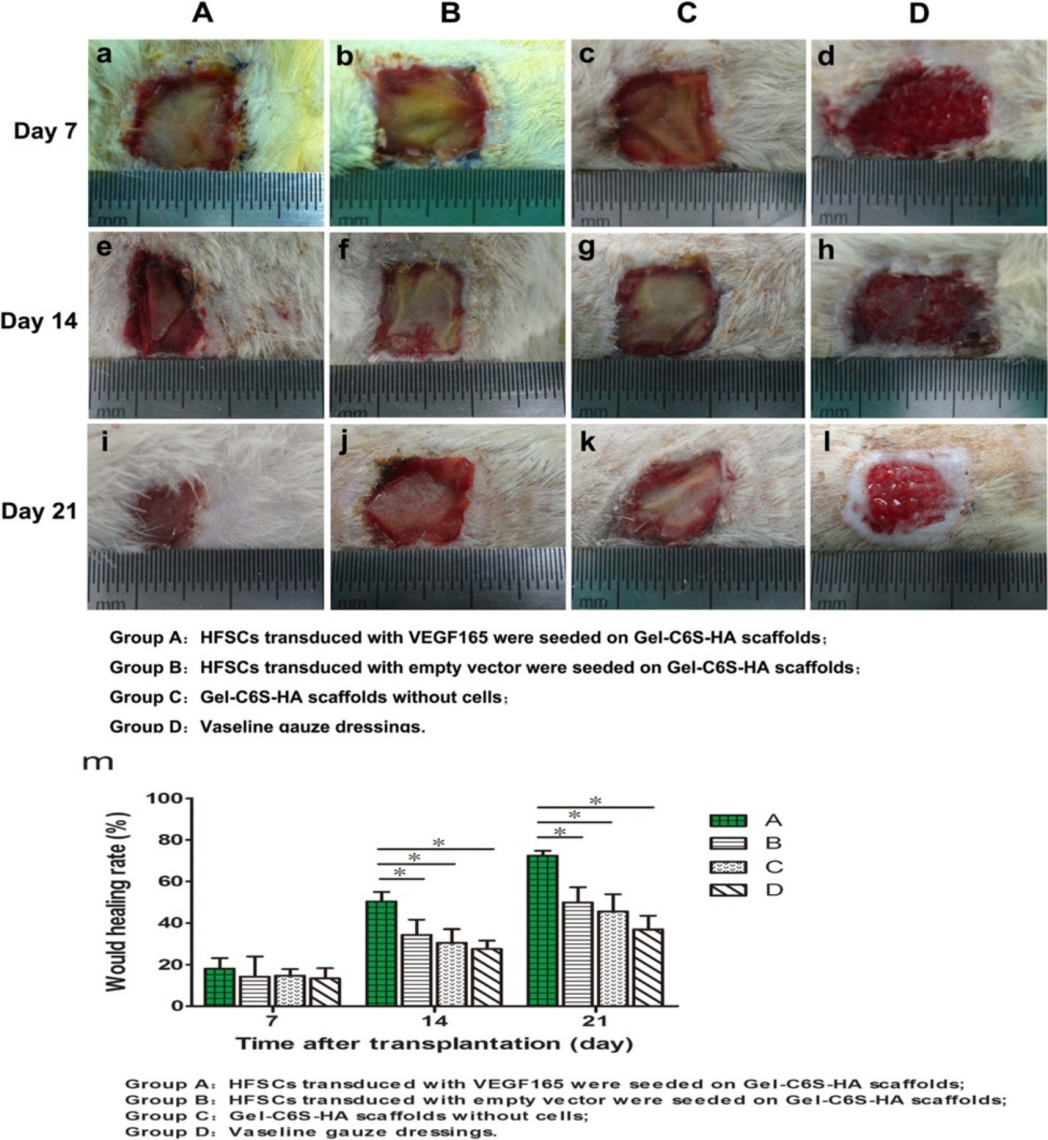
**Postoperative skin wound. (a)** to **(l)** After 7, 14 and 21 days, there were no visible signs of wound inflammation in all four groups, the graft was in close contact with the wound, and the wound of Group D was dry and clean with red granulation. **(m)** Wound healing rates of Groups A to C were fast after 7 days, with the graft absorption speed in Group A being fastest at 14 and 21 days. In Group A, the graft combined solidly with its surrounding tissue, and the wound healing rate was significantly higher than in other groups. *P <0.05. Gel-C6S-HA, gelatin–chondroitin-6-sulfate–hyaluronic acid; HFSC, hair follicle stem cell; VEGF, vascular endothelial growth factor.

After 7 days, there was no significant swelling, exudate or infection observed for all groups. In all cases, the transplant was in close contact with the wound. For Group D, the wound was dry and had red granulation (Figure 6a,b,c). After 14 and 21 days, the wound area of Group A was dry and clean. This construct resulted in the fastest absorption and was combined solidly with the surrounding wound. Furthermore, the wound healing rate was significantly faster compared with the other groups (Figure 6d,e,f,g,h,i,j,k,l). Meanwhile, we analyzed rat wound healing rates at 7, 14 and 21 days (Figure 6m); after 14 and 21 days, a statistically significant difference was observed between the experimental group (Group A) and the other three groups (Groups B to D, *P* <0.05). After 21 days, the wound healing rate in Group A was 1.3-fold, 1.65-fold and 1.96-fold higher than in Groups B, C and D, respectively.

### Hematoxylin and eosin staining of the transplanted scaffold

**Figure 7:**
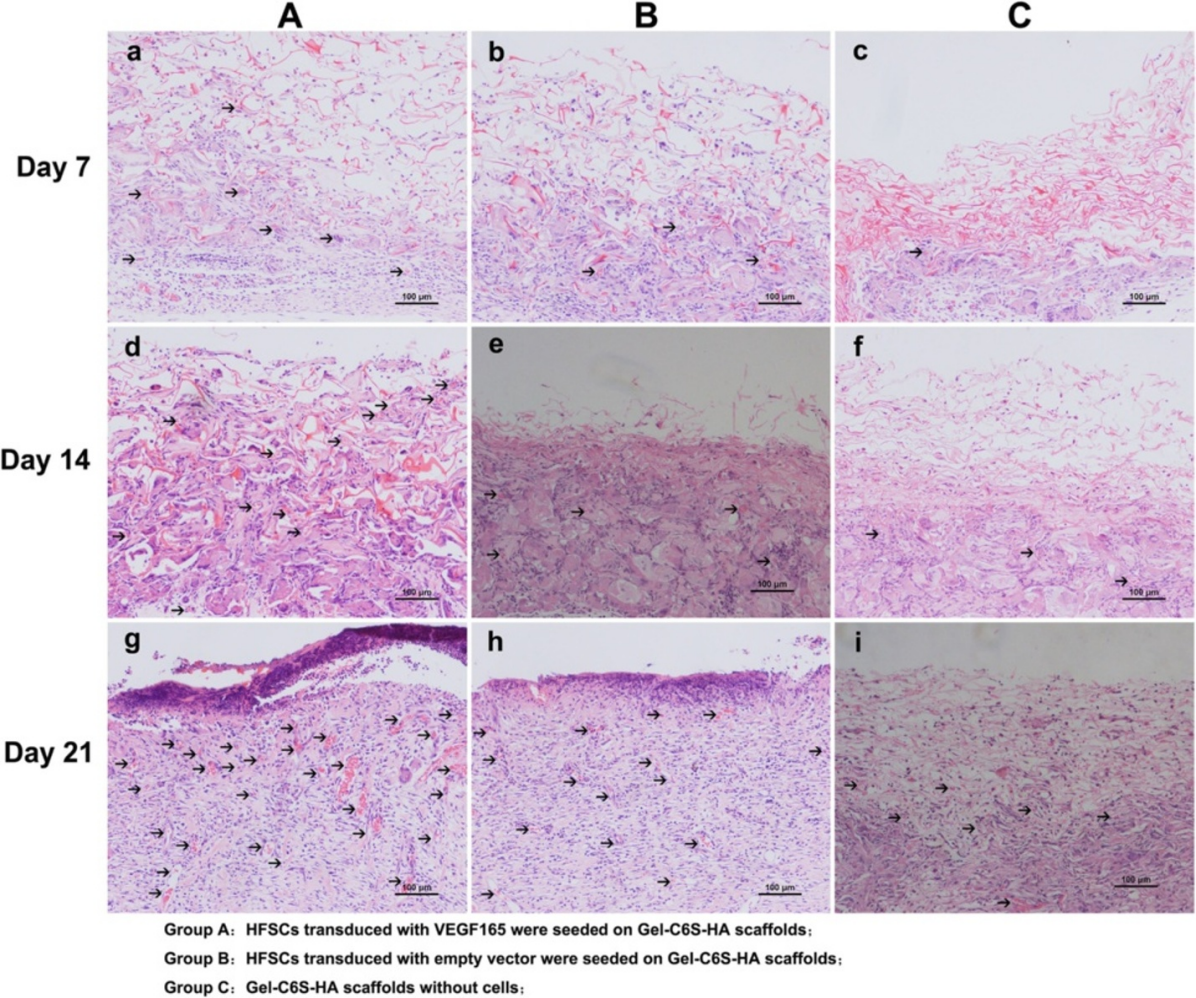
**Hematoxylin and eosin staining of the graft. (a)**, **(b)**, **(c)** After 7 days, skin grafts in Groups A and B had formed microvessels, the three-dimensional morphology of the scaffold was loosely structured and cell distribution was uniform; conversely, the Group C scaffold was clear. **(d)**, **(e)**, **(f)** After 14 days, the newly formed vessels in Group A were significantly increased with relatively fewer in Group B. Scaffolds in Group A and B were full of uniformly distributed cells, with varying degrees of degradation and absorption. Subcutaneous tissue cells tended to migrate into the Group C scaffold material, but numbers remained limited. **(g)**, **(h)**, **(i)** After 21 days, new blood vessels with uniform distribution could be found within the full layer of Group A, and these vessels were large and abundant. Vascularization in Group B was different from that in Group A, with only a few blood vessels formed at the junctions between the subcutaneous tissue and scaffold in Group C. Scale bars: 100 μm. Gel-C6S-HA, gelatin–chondroitin-6-sulfate–hyaluronic acid; HFSC, hair follicle stem cell; VEGF, vascular endothelial growth factor.

After 7 days, small microvessels were generated in the transplanted scaffold in both Group A and Group B (Figure 7a,b). The three-dimensional scaffold structure was loosely structured with uniform cell distribution, while the trestle structure of Group C was compact (Figure 7c), with a small number of cells aggregated at the subcutaneous junctions. After 14 days, Group A and Group B were observed to have good scaffold in-fill of cells; however, the scaffolds had different levels of absorption (Figure 7d,e). Newly generated blood vessels were significantly increased in Group A. In comparison, subcutaneous tissue cells continued migrating into the scaffold; however, numbers were limited in Group C. After 21 days, part of the epidermis appeared to undergo epidermalization in Group A (Figure 7g). This was observed within the whole layer, and a large number of new vessels with homogeneous distribution were observed. The degree of vascularization in Group B was not comparable with that of the experimental group (Group A; Figure 7h), and only a few vessels were seen in Group C (Figure 7i).

### Immunohistochemical staining and microvessel density count

**Figure 8:**
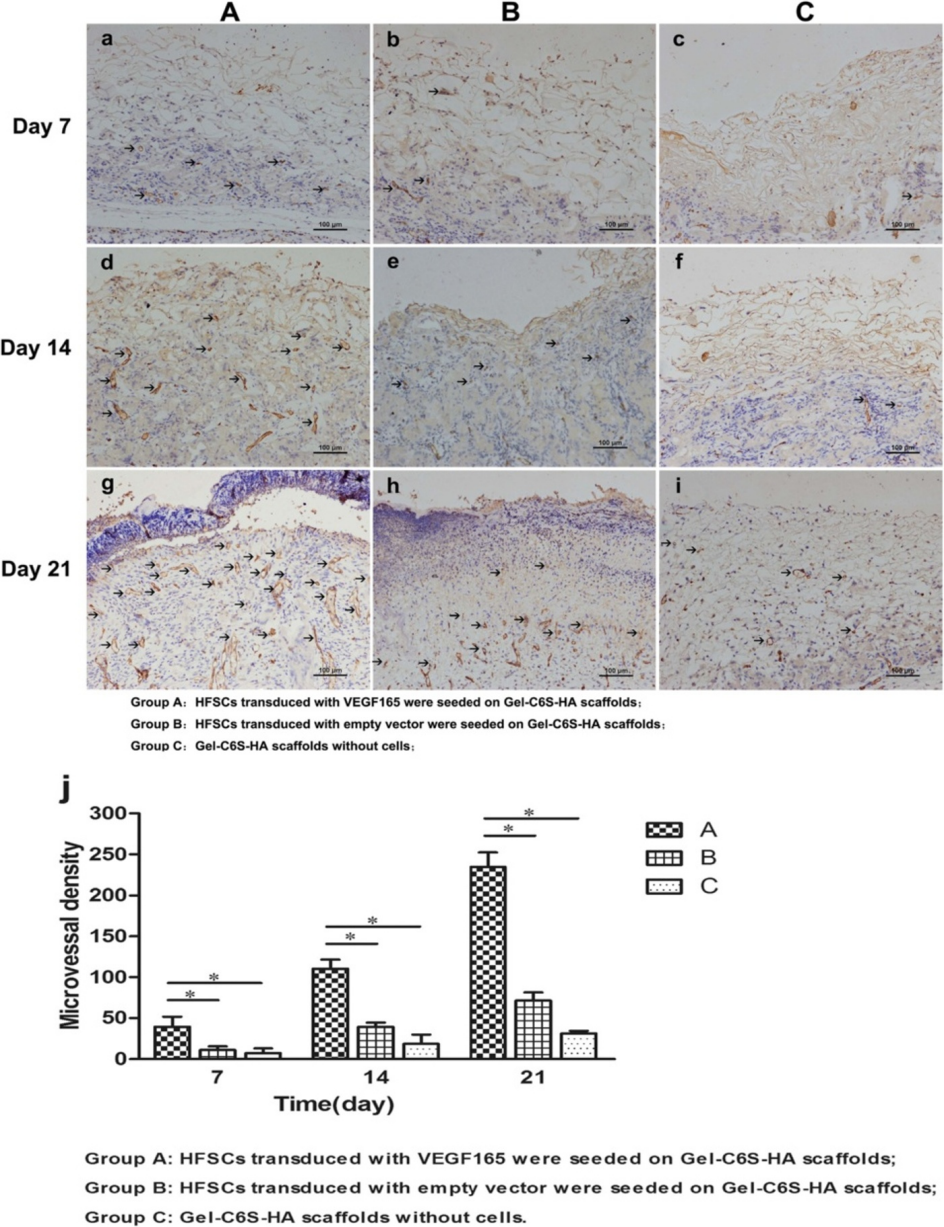
**Markers of new blood vessels. (a)**, **(d)**, **(g)** At each time point after surgery, positive expression (brown) of CD31 in Group A was most abundant. **(b)**, **(e)**, **(h)** In Group B, CD31 expression was lower than Group A, and the vessels were relatively small. **(c)**, **(f)**, **(i)** In Group C, CD31 was significantly lower than that in Groups A and B, with only trace expression. **(j)** Vessel density results. Scale bars: 100 μm. Group A was compared with Groups B and C, *P <0.05. Gel-C6S-HA, gelatin–chondroitin-6-sulfate–hyaluronic acid; HFSC, hair follicle stem cell; VEGF, vascular endothelial growth factor.

**Figure 9:**
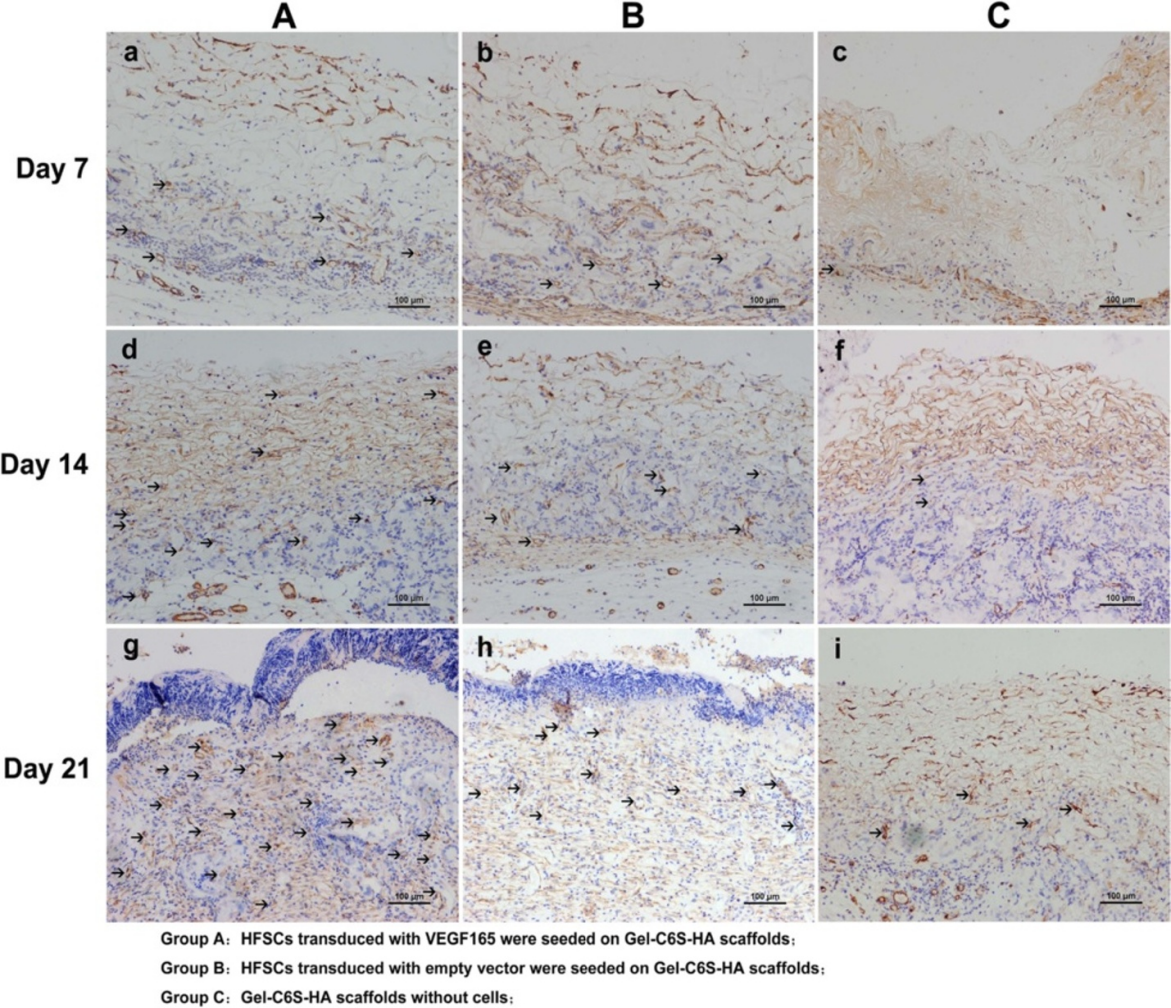
**Markers of mature blood vessels. (a)**, **(d)**, **(g)** At each time point after surgery, vessels were larger and uniformly distributed with the highest vascular maturity in Group A. **(b)**, **(e)**, **(h)** Mature vessels were smaller in Group B. **(c)**, **(f)**, **(i)** There were almost no vessels in the upper layer of the scaffold in Group C, and the positive expression (brown) zone was confined to the junction of the subcutaneous tissue. There were more blood vessels growing into the scaffold when it was closer to the subcutaneous tissue, but the number remained small and fewer than in the experimental group (Group A). Scale bars: 100 μm. Gel-C6S-HA, gelatin–chondroitin-6-sulfate–hyaluronic acid; HFSC, hair follicle stem cell; VEGF, vascular endothelial growth factor.

At each time point after surgery, CD31-positive expression was significantly higher in Group A than in Groups B and C (Figure 8a,d,g). Furthermore, it was found using α-SMA that the vascular morphology of Group A was largest with the highest blood vessel maturation (Figure 9a,d,g). The number of new blood vessels in Group B was second highest (Figure 8b,e,h) but with relatively small blood vessels (Figure 9b,e,h). In the Group C scaffold, CD31 expression was negligible (Figure 8c,f,i) with only a trace of α-SMA expression (Figure 9c,f,i). Furthermore, CD31-positive expression was confined within the scaffold and the contacting zone of the subcutaneous tissue. At the nearside to the subcutaneous tissues, the number of blood vessels growing into the rat body was more abundant, but to a lesser extent compared with the experimental group (Group A). Furthermore, after 21 days in Groups A and B, different levels of epidermalization appeared and the cells in the epidermis were highly aggregated.

Cell counts using Image Pro Plus 6.0 software found after 7, 14 and 21 days that MVD counts in Group A were 36.7 ± 11.9, 110.3 ± 11.3 and, 234.7 ± 17.8/mm^2^, respectively. These counts were significantly higher than in Groups B and C. MVD counts in Group B were 11.0 ± 4.7, 39.3 ± 4.9 and 71.3 ± 10.0/mm^2^, respectively, while in Group C they were lowest at 7.3 ± 5.8, 18.7 ± 11.4 and 31.3 ± 3.4/mm^2^. After 21 days, the MVD of Group A was 3.29-fold and 7.49-fold greater than Group B and Group C, respectively. At each time point, there was a statistically significant difference (*P* <0.05) between Group A and Groups B and C (Figure 8j).

### Immunogenicity results

**Figure 10:**
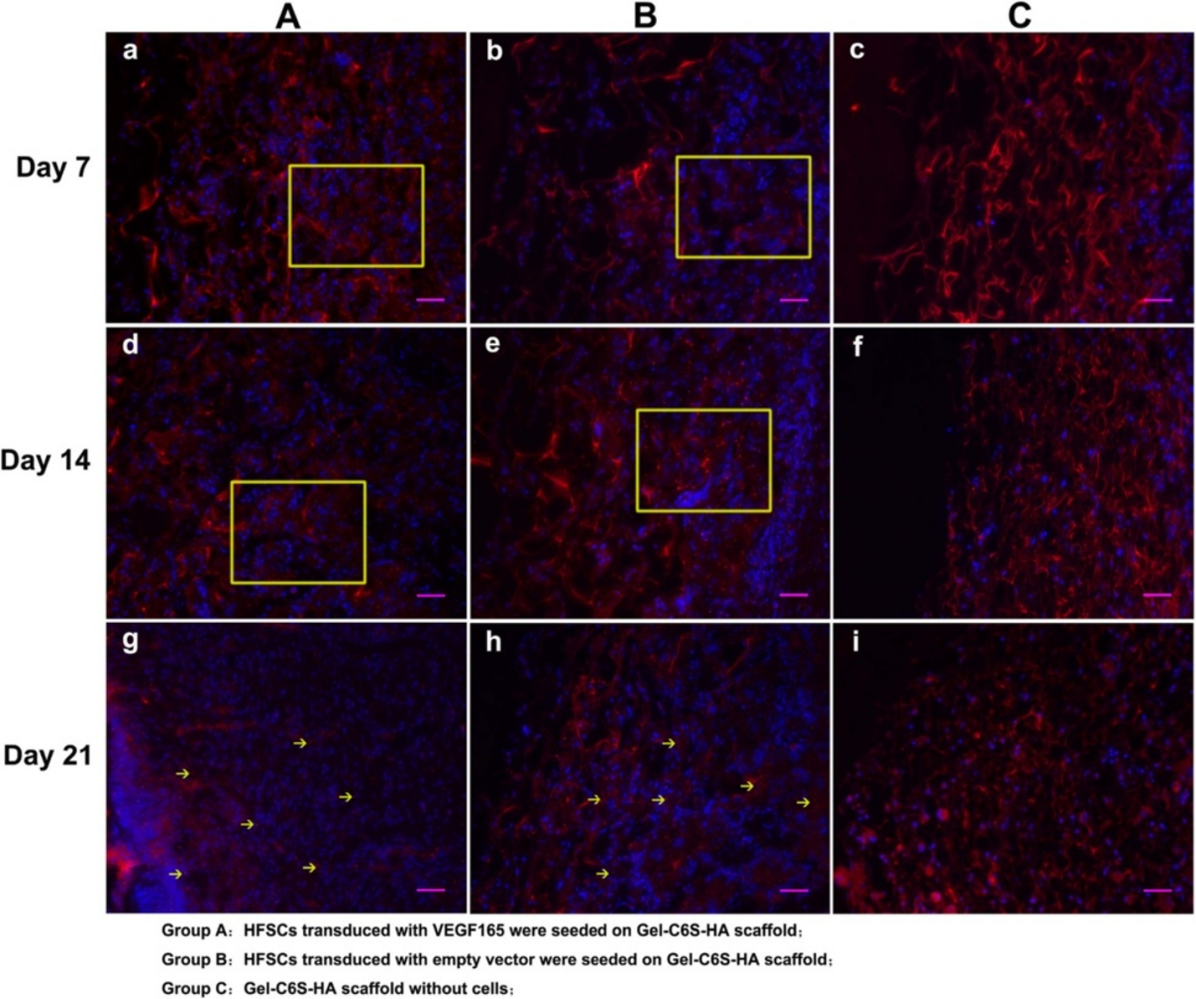
**Major histocompatibility complex class I immunofluorescence staining.** Yellow boxes and arrows highlight major histocompatibility complex class I (MHC-I) antibody expressed on the cytomembrane. Within the 21 days, almost no significant difference was detected in the expression of MHC-I antibodies in the three groups. **(a)**, **(b)**, **(d)**, **(e)** There were a few red dots within the first 14 days in Groups A and B. **(c)**, **(f)**, **(i)** Postoperative at 21 days, there was no visible MHC-I expression in all cells migrating into the scaffold in Group C. Scale bars: 50 μm. Gel-C6S-HA, gelatin–chondroitin-6-sulfate–hyaluronic acid; HFSC, hair follicle stem cell; VEGF, vascular endothelial growth factor.

During the experimental period, there was no skin wound redness, exudate, infection or other changes observed in Groups A to C, and all transplanted tissue-engineered skin remained viable (Figure 6). As shown by MHC-I antibody immunofluorescence staining, a minority of cells expressed a trace of red fluorescence in the three-dimensional skin transplanted scaffolds (Figure 10). Within the 21 days, almost no significant difference was detected in the expression of MHC-I antibodies in Groups A, B and C. Based on enlarged images and careful observation, we can find that there were a little bit of red dots within the first 14 days in Groups A and B (Figure 10a,b,d,e). Expression of MHC-I was absent from cells in the groups.

## Discussion

In the past 20 years, research and development on skin tissue engineering has made great progress, and the effective use of tissue-engineered skin to treat large skin defects has become a hot topic of study, now considered one of the best treatment approaches to repair skin defects [31, 32]. However, even with increased research, the occurrence of clinically applied tissue-engineered skin is low. One of the main reasons for this is the difficulty in vascularization of the tissue-engineered skin.

In this study, Gel-C6S-HA scaffolds combined with VEGF165 gene-modified rHFSCs were used as skin substitutes. Cell-seeded scaffolds were transplanted in a rat model with a full-layer thickness skin defect, to investigate the role and influence of the construct on vascularization of tissue-engineered skin at different time points.

During the continual development of skin tissue engineering approaches, stem cells are a frequently investigated cell source; however, this is not without limitations. Embryonic stem cells, mesenchymal stem cells, epidermal stem cells and HFSCs have all been reported as useful tissue engineering cell sources, and each has its own advantages and disadvantages. Embryonic stem cells have an extensive differentiation capacity; however, ethical and legal problems exist in harvesting and applying this cell source, which severely limits its therapeutic use [33, 34]. Mesenchymal stem cells are easily isolated and cultured, have strong proliferative capacity and low immunogenicity. Although mesenchymal stem cells can be induced to differentiate into epidermal cells and fibroblasts, limitations include donor site morbidity and low harvest volume [35, 36]. Epidermal stem cells can form tissue-engineered skin with hair follicles, sweat glands and other subsidiary organs, but again problems exist in using this cell type. Often, the new and large area of wound from autologous materials results in a serious allograft immune rejection reaction [37, 38]. HFSCs are reported to have a strong proliferative capacity and potential to differentiate towards full-thickness layer skin cells. With such an abundant source of hair follicles and convenience in obtaining the cells with minimal trauma, HFSCs appear to be an ideal cell source for tissue-engineered skin [25–27, 39, 40]. HFSCs exist predominantly in the hair follicle bulge and have fast and strong adhesion characteristics [21–23, 41]. Using a mixed enzyme digestion, microdissection and a differential adherence sorting method (Figure 2a,b,c,d,e,f), HFSCs with a strong proliferative capacity were obtained in this study (Figure 2g,h). Interestingly, when applied for 21 days *in vivo*, the transplanted materials in the groups with inoculated rHFSCs had different degrees of epidermalization tendency, while the scaffold without rHFSCs did not result in epidermalization. We speculate that HFSCs secrete a variety of cytokines to promote wound healing, including epidermal growth factor and basic fibroblast growth factor, to name a few. When implanted in an internal environment *in vivo*, these cells probably differentiate into epidermal cells and play a role in promoting early vascularization of the wound.

Key requirements for tissue-engineered skin include living cells and a suitable scaffold material for cell growth interactions. Current skin substitutes do not have vascular structures or a source of nutrition themselves, and blood vessels from the surrounding tissues are required to grow into them to create a loop, thus providing nutrition after transplantation. In this study, we used gelatin, chondroitin-6-sulfate and hyaluronic acid as an extracellular matrix for biomimetic skin cells. Following cell inoculation into the Gel-C6S-HA scaffolds, cells were uniformly diffused into the scaffold pores, and adhered and migrated along the scaffold structure in a timely manner to grow three-dimensional tissue (Figure 4a,b,c,d,e,f). Previous studies on scaffold structure have shown that during the early period after transplantation, before microcirculation is established between the tissue-engineered skin and wound, cells in the transplanted materials beyond 200 μm usually die in absence of adequate nutritional support [42]. The three-dimensional structure of the scaffold could therefore significantly improve the communication rate and water permeability (Figure 1) to meet requirements, by ensuring high porosity and high surface area to enhance cell adhesion and migration, thus promoting vascularization of the new skin [43–45]. In our study, we found no significant difference in rHFSC morphology, adhesion and proliferative capacity on Gel-C6S-HA scaffolds compared with the control group (Figure 4g).

The formation of newly formed blood vessels within tissue-engineered skin substitutes is a key factor in evaluating their vascularization capability. CD31, as a transmembrane protein, is a good marker of endothelial cells and can be used to identify newly formed blood vessels owing to their high sensitivity and specificity [46, 47]. α-SMA is expressed primarily in the vascular middle layer, specifically identifying smooth muscle cells surrounding endothelial cells, and can be used to determine the level of maturity of newborn blood vessels and to identify mature vessels [48]. By applying immunohistochemical staining and MVD counting to our *in vivo* studies, we found that post-treatment positive expression of both CD31 and α-SMA reached a maximum in Group A. Group A also had the greatest number of new blood vessels formed and the most blood vessel maturation. Smaller blood vessels were formed in Group B; however, their total was relatively low with a small size in comparison. The three-dimensional characteristics of these two skin grafts (Groups A and B) were loosely structured with uniform cell distribution. Conversely, the scaffold pore structures in Group C were compactly structured and a small number of cells aggregated at the subcutaneous junctions and migrated into the scaffold. Only the subcutaneous tissue had a small amount of mature blood vessels growing into the scaffold (Figures 8 and 9). In our study, we have shown VEGF165 stable-expressing HFSCs play an important role, by significantly enhancing the amount of VEGF165 protein short term, to create a high VEGF165 level repair microenvironment. This in turn improves and promotes the early vascularization process of skin substitutes, thus facilitating wound healing.

Immune rejection is still a key factor to consider when transplanting tissue-engineered skin. Expression of MHC-I can be used as an important immune rejection marker during the early period of tissue-engineered skin transplantation [49, 50]. In our study, there were a few red dots within the first 14 days in Groups A and B (Figure 10a,b,d,e). We speculate that these tiny particles in the boxes and arrows in yellow might represent a staining artifact that was caused by experimental operation. Radically speaking, there is no obvious expression of MHC-I. Following observation and analysis of the wound after surgery, significant swelling, exudate and infection were absent in all groups (Figure 6); the level of immune rejection was therefore acceptable and would permit survival of the transplanted skin substitutes.

## Conclusions

After transplantation of VEGF165 gene-modified rHFSCs, seeded on three-dimensional Gel-C6S-HA constructs, into full-thickness layer skin defects, the VEGF165 level in the repair microenvironment was increased. This in turn significantly improved the partial revascularization ability of the tissue-engineered skin, thus promoting wound healing. This tissue-engineered skin strategy has excellent feasibility and efficacy, providing an important theoretical basis for further research and development of skin replacement constructs, with a view to their future clinical application.

## Abbreviations

CK: cytokeratin
Gel-C6S-HA: gelatin–chondroitin-6-sulfate–hyaluronic acid
HFSC: hair follicle stem cell
MHC-I: major histocompatibility complex class I
MVD: microvessel density
P: passage
PBS: phosphate-buffered saline
PCS: polymerase chain reaction
rHFSC: rat hair follicle stem cell
α-SMA: alpha smooth muscle actin
VEGF: vascular endothelial growth factor.
